# OXA-484, an OXA-48-Type Carbapenem-Hydrolyzing Class D β-Lactamase From *Escherichia coli*

**DOI:** 10.3389/fmicb.2021.660094

**Published:** 2021-05-12

**Authors:** Julian Sommer, Kristina M. Gerbracht, Felix F. Krause, Florian Wild, Manuela Tietgen, Sara Riedel-Christ, Janko Sattler, Axel Hamprecht, Volkhard A. J. Kempf, Stephan Göttig

**Affiliations:** ^1^Institute for Medical Microbiology and Infection Control, University Hospital, Goethe University Frankfurt am Main, Frankfurt, Germany; ^2^Faculty of Biological Sciences of the Goethe University Frankfurt am Main, Frankfurt, Germany; ^3^University Center of Competence for Infection Control of the State of Hesse, Frankfurt, Germany; ^4^Institute for Medical Microbiology, Immunology and Hygiene, German Center for Infection Research (DZIF Partner Site Cologne-Bonn), University Hospital of Cologne, Cologne, Germany; ^5^Institute for Medical Microbiology, University Hospital of Oldenburg, Oldenburg, Germany

**Keywords:** OXA-48, OXA-484, IncX, carbapenemases, beta-lactamases, *Enterobacterales*, plasmid

## Abstract

OXA-48-like carbapenemases are among the most frequent carbapenemases in Gram-negative *Enterobacterales* worldwide with the highest prevalence in the Middle East, North Africa and Europe. Here, we investigated the so far uncharacterized carbapenemase OXA-484 from a clinical *E. coli* isolate belonging to the high-risk clone ST410 regarding antibiotic resistance pattern, horizontal gene transfer (HGT) and genetic support. OXA-484 differs by the amino acid substitution 214G compared to the most closely related variants OXA-181 (214R) and OXA-232 (214S). The *bla*_OXA__–__484_ was carried on a self-transmissible 51.5 kb IncX3 plasmid (pOXA-484) showing high sequence similarity with plasmids harboring *bla*_OXA__–__181_. Intraspecies and intergenus HGT of pOXA-484 to different recipients occurred at low frequencies of 1.4 × 10^–7^ to 2.1 × 10^–6^. OXA-484 increased MICs of temocillin and carbapenems similar to OXA-232 and OXA-244, but lower compared with OXA-48 and OXA-181. Hence, OXA-484 combines properties of OXA-181-like plasmid support and transferability as well as β-lactamase activity of OXA-232.

## Introduction

Carbapenem resistance in *Enterobacterales* has dramatically increased worldwide in recent years and poses a major threat to public health. Antibiotic treatment options for infections caused by carbapenem-resistant *Enterobacterales* (CPE) are severely limited, with often very few or even no antibiotic agents remaining effective. Resistance to carbapenems is primarily caused by carbapenemases, i.e., bacterial enzymes that hydrolyze carbapenems and most other β-lactam antibiotics. OXA-48 is one of the most prevalent carbapenemases worldwide with the highest prevalence in the Middle East, North Africa and Europe (Cantón et al., 2012). OXA-48 enzymes lead to carbapenem resistance and are most often found in *Escherichia coli* and *Klebsiella pneumoniae* (Pitout et al., 2019).

The rapid dissemination of OXA-48 has been attributed to efficient horizontal gene transfer (HGT) of plasmids harboring *bla*_OXA__–__48_ and a low fitness burden by carriage of these plasmids (Hamprecht et al., 2019). At least 35 variants of OXA-48, commonly referred to as OXA-48-like β-lactamases (e.g., OXA-181, OXA-232, OXA-244), have been reported (Naas et al., 2017). Even though these variants differ only by single or few amino acid substitutions, a high degree of diversity is observed regarding the β-lactam hydrolysis pattern, genetic support of OXA-48-like encoding genes and HGT (Potron et al., 2011, 2013). The gene encoding OXA-48, *bla*_OXA__–__48_, is mostly present on an epidemic 63.6 kb IncL plasmid and bracketed by two identical insertion sequences (IS), IS*1999*, forming the composite transposon Tn*1999* (Poirel et al., 2012). In contrast, *bla*_OXA__–__181_ is primarily found on a 51.5 kb IncX3 plasmid and has been associated with IS*Ecp1* in a Tn*2013* transposon structure, whereas *bla*_OXA__–__232_ is commonly located on a non-conjugative 6.1 kb ColE-type plasmid (Qin et al., 2018; Li et al., 2019). In case of *bla*_OXA__–__244_, the gene is mostly identified on the chromosome of *E. coli* and has been increasingly identified in the European Union in recent years (European Centre for Disease Prevention and Control, 2020; Kremer et al., 2020; Chudejova et al., 2021).

OXA-484, a variant of the OXA-48-like family, has been previously reported from a collection of OXA-48-like carbapenemase in the United Kingdom, but so far only the sequence is known (Findlay et al., 2017). Here we report on the genetic background, antibiotic resistance phenotype and HGT of this carbapenemase.

## Materials and Methods

### Bacterial Isolates and Antimicrobial Susceptibility Testing

*Enterobacterales* clinical isolates carrying the plasmid-borne OXA-48-like carbapenemases OXA-48, OXA-181, OXA-232, OXA-244, and OXA-484 were recovered from patients of the University Hospital Frankfurt, Germany (Table 1). All isolates were phenotypically characterized and tested for *bla*_OXA__–__48__–__like_ by PCR and Sanger sequencing as previously described (Göttig et al., 2015). Antimicrobial susceptibility was determined using antibiotic gradient strips (Liofilchem, Roseto degli Abruzzi, Italy), broth micro dilution for colistin and agar dilution for fosfomycin as recommended by EUCAST. Minimal inhibitory concentrations (MIC) were interpreted according to EUCAST guidelines v10.0. For detection of carbapenemases, chromID^®^ CARBA SMART plates (bioMérieux, Nürtingen, Germany) and the immunochromatographic CARBA 5 lateral flow test (NG-Biotech, Guipry, France) were used (Baeza et al., 2019; Göttig et al., 2020).

**TABLE 1:** Characteristics of clinical isolates harboring OXA-48-like carbapenemases and recipient strains *E.coli* J53 and *K. quasipneumoniae* subsp. *quasipneumoniae* PRZ.

Isolate	Species	OXA-48-like	ST^a^	Plasmid size (kb)	Inc^b^	References
EC-JS316	*E. coli*	OXA-484	410	51.5	IncX3	This study
EC-2700	*E. coli*	OXA-48	354	61.1	IncL	Hamprecht et al., 2019
EC-JS426	*E. coli*	OXA-48	448	63.6	IncL	This study
KP-1402	*K. pneumoniae*	OXA-48	16	63.6	IncL	This study
KP-1673	*K. pneumoniae*	OXA-48	101	63.6	IncL	Hamprecht et al., 2019
KP-4113	*K. pneumoniae*	OXA-232	231	6.1	ColE	This study
KP-4814	*K. pneumoniae*	OXA-232	2096	6.1	ColE	This study
EC-5255	*E. coli*	OXA-181	3268	51.5	IncX3	This study
EC-2800	*E. coli*	OXA-181	940	51.5	IncX3	This study
KP-4313	*K. pneumoniae*^c^	OXA-181	5040	50.2	IncX3	This study
KP-SC771	*K. pneumoniae*	OXA-181	307	51.5	IncX3	This study
EC-SC1140	*E. coli*	OXA-244	38	–	–	This study
J53	*E. coli*	–	10	–	–	Gruber et al., 2014
PRZ	*K. pneumoniae*^d^	–	5208	–	–	Gruber et al., 2014

**^*a*^MLST according to Oxford scheme for E. coli and Pasteur scheme for K. pneumoniae. ^*b*^Plasmid support of bla_OXA__–__48__–__like_. ^*c*^K. quasipneumoniae subsp. similipneumoniae. ^*d*^K. quasipneumoniae subsp. quasipneumoniae.**

### Cloning of *bla*_OXA__–__48__–__like_

The open reading frames of *bla*_OXA__–__484_, *bla*_OXA__–__244_, *bla*_OXA__–__232_, *bla*_OXA__–__181_, and *bla*_OXA__–__48_, including their native promoters, were amplified using the primers OXA-TOPO-F1, OXA-244-TOPO-F1, OXA-48-TOPO-F1, and preOXA-48B (Supplementary Table 1) and cloned into the expression vector pCR-Blunt II-TOPO (Invitrogen, Darmstadt, Germany) as described before (Potron et al., 2011). The resulting vectors pTOPO_OXA-484, pTOPO_OXA-244, pTOPO_OXA-232, pTOPO_OXA-181, and pTOPO_OXA-48 were used to transform electrocompetent *E. coli* J53 and *K. quasipneumoniae* subsp. *quasipneumoniae* PRZ (Choi et al., 2006).

### Horizontal Gene Transfer of *bla*_OXA__–__48__–__like_ Harboring Plasmids

Transconjugation of *bla*_OXA__–__48__–__like_ harboring plasmids was conducted by liquid mating as previously described (Gruber et al., 2014). Briefly, a mixture of donors and recipients in brain heart infusion broth were incubated over night at 37°C and subsequently plated on chromogenic agar plates containing 100 mg/L sodium azide and 10 mg/L amoxicillin-clavulanic acid to select for transconjugants (Tc). Clinical *E. coli* and *K. pneumoniae* isolates encoding *bla*_OXA__–__48_, *bla*_OXA__–__181_, *bla*_OXA__–__232_, or *bla*_OXA__–__484_ were employed as donors and sodium azide-resistant *E. coli* J53 and *K. quasipneumoniae* subsp. *quasipneumoniae* PRZ as recipients (Hamprecht et al., 2019). Plasmid DNA from clinical isolates carrying *bla*_OXA__–__232_ was extracted using the PureYield^TM^ Plasmid Midiprep System (Promega, Walldorf, Germany). Transformation of J53 and PRZ using *bla*_OXA__–__232_ encoding plasmids was done by electroporation and transformants (Tf) were selected as described above. Presence of OXA-48-like carbapenemases in Tc and Tf was verified by disk diffusion antibiotic testing and the CARBA 5 lateral flow test. All *E. coli* J53 Tc were analyzed using long-read genome sequencing and disk diffusion testing as recommended by EUCAST. Transconjugation frequency was determined by dividing the numbers of Tc colonies by the number of acceptor colonies.

### Whole Genome Sequencing

Whole genome sequencing was carried out for all clinical isolates using short-read technology (MiSeq or NovaSeq platform, Illumina, San Diego, United States) and long-read technology (MinION platform, Oxford Nanopore Technologies, Oxford, United Kingdom). DNA was extracted from isolates using the DNeasy UltraClean Microbial Kit (Qiagen, Hilden, Germany). For Illumina sequencing, a v3 reagent kit was applied generating either 150 or 250 bp paired-end reads. Library preparation for Nanopore sequencing was done using the SQK-RBK004 rapid barcoding kit. Sequencing was performed on a MinION MK1B sequencer utilizing a R9.4.1 flow cell. Raw signal data was base called and demultiplexed using the high accuracy base calling model of guppy basecaller version 4.0.11.

### Bioinformatic Analysis

Raw data was filtered using trimmomatic for short reads and NanoFilt for long reads resulting in datasets of reads with an average genome coverage of at least 100-fold for short-reads and at least 30-fold for long-reads (Bolger et al., 2014; de Coster et al., 2018). *De novo* hybrid assembly was conducted using Unicycler version 0.4.8 utilizing the bold assembly mode (Wick et al., 2017). The obtained assemblies were annotated using Prokka version 1.14.6 (Seemann, 2014). Sequence types were determined using the software mlst^1^ v2.19.0 (Carattoli et al., 2014). ABRicate^2^ v1.0.1 was applied using the databases PlasmidFinder and NCBI AMRFinderPlus for identification of plasmid incompatibility groups and antibiotic resistance genes, respectively, using thresholds of 100% gene coverage and ≥98% nucleotide sequence identity (Feldgarden et al., 2019). ISfinder was employed for annotation of insertion sequences (Siguier et al., 2006). Plasmid sequences were aligned using MAFFT 1.4.0 and visualized using Geneious^3^ 11.1.5 (Katoh and Standley, 2013).

### Statistical Analysis

For the comparison of transconjugation frequencies, continuous variables were assessed by Mann-Whitney *U* test. A *P*-value of < 0.05 was considered significant.

## Results

### Identification of a Clinical Isolate Harboring *bla*_OXA__–__484_

The *E. coli* isolate JS316 (EC-JS316) was obtained from a rectal swab of a patient admitted to the University Hospital Frankfurt in Germany. The patient was hospitalized due to acute dengue fever following a stay in India. The isolate EC-JS316 showed resistance to ertapenem with a MIC of 1 mg/L but was susceptible to imipenem (MIC 0.25 mg/L) and meropenem (MIC 0.125 mg/L) (Table 2). Furthermore, the isolate was resistant to piperacillin-tazobactam, cefotaxime, fluoroquinolones, gentamicin, tobramycin and trimethoprim-sulfamethoxazole, but remained susceptible to amikacin, fosfomycin, tigecycline and colistin (Supplementary Table 2). Strikingly, the isolate EC-JS316 did not grow on chromID^®^ CARBA SMART screening agar plates used for detection of CPE.

**TABLE 2:** MICs of the clinical isolate EC-JS316 and transformants (Tf) or transconjugants (Tc) of the recipients *E. coli* J53 and *K. quasipneumoniae* subsp. *quasipneumoniae* PRZ harboring plasmids encoding *bla*_OXA__–__484_, *bla*_OXA__–__181_, *bla*_OXA__–__232_, and *bla*_OXA__–__48_.

	MIC (mg/L)										
β-Lactam^a,b,c^	EC-JS316	J53	Tc pOXA-484 J53^1^	Tf pOXA-232 J53^2^	Tc pOXA-181 J53^3^	Tc pOXA-48 J53^4^	PRZ	Tc pOXA-484 PRZ^1^	Tf pOXA-232 PRZ^2^	Tc pOXA-181 PRZ^3^	Tc pOXA-48 PRZ^4^
Ampicillin	>256	2	>256	>256	>256	>256	64	>256	>256	>256	>256
Amoxicillin+CLA	128	2	>256	>256	>256	>256	2	>256	>256	>256	>256
Ampicillin+SUL	>256	2	>256	>256	>256	>256	8	>256	>256	>256	>256
**Piperacillin**	>256	2	>**256**	>**256**	**32**	**64**	8	>256	>256	>256	>256
**Piperacillin+TAZ**	>256	1	**128**	>**256**	**32**	**64**	8	256	>256	>256	>256
Ticarcillin+CLA	>256	4	>256	>256	>256	>256	16	>256	>256	>256	>256
Cephalothin	>256	8	16	16	16	16	4	16	32	16	16
Cefuroxime	>256	4	8	8	8	8	4	4	8	4	8
Cefoxitin	64	4	4	4	4	4	4	4	4	4	4
Cefotaxime	4	0.06	0.12	0.25	0.25	0.25	0.06	0.25	0.5	0.5	0.5
Ceftazidime	2	0.06	0.25	0.25	0.25	0.12	0.03	0.25	0.5	0.25	0.25
**Temocillin**	128	4	**32**	**64**	**256**	**256**	2	**32**	**64**	**256**	**256**
Ertapenem	1	0.03	0.12	0.12	0.12	0.25	0.01	1	2	2	2
Imipenem	0.25	0.25	0.5	0.5	1	1	0.12	0.5	0.5	1	1
Meropenem	0.12	0.03	0.06	0.06	0.12	0.12	0.03	0.12	0.12	0.25	0.25
Doripenem	0.06	0.01	0.06	0.06	0.12	0.12	0.03	0.06	0.12	0.12	0.12
Aztreonam	1	0.03	0.06	0.06	0.03	0.03	0.01	0.06	0.06	0.03	0.03

**^*a*^CLA, clavulanic acid (2 mg/L). ^*b*^SUL, sulbactam (4 mg/L). ^*c*^TAZ, tazobactam (4 mg/L). pOXA-48-like plasmid donors: ^1^EC-JS316, ^2^KP-4814, ^3^EC-2800, ^4^EC-2700. MICs of Tc and Tf with at least 2-fold differences compared to OXA-484 are highlighted in bold.**

The CARBA 5 lateral flow test revealed the presence of an OXA-48-like β-lactamase. PCR and subsequent Sanger sequencing of the *bla*_OXA__–__48__–__like_ open reading frame identified *bla*_OXA__–__484_ (accession no. NG_049766.1). The gene *bla*_OXA__–__484_ has a single nucleotide substitution at position 640 (A→G) compared to the most closely related gene *bla*_OXA__–__181_ and two substitutions at position 639 (A→G) and 641 (T→A) compared to *bla*_OXA__–__232_. These substitutions result in different amino acids at position 214 which is essential for enzyme activity: 214G for OXA-484, 214R for OXA-181, and 214S for OXA-232. The amino acid change R214S is also present in OXA-244 (Supplementary Figure 1).

### Genome Analysis of *E. coli* EC-JS316

The genome of EC-JS316 was sequenced and assembled using a combined short- and long-read sequencing approach, resulting in the nucleotide sequences of a circular chromosome and five circular plasmids (Supplementary Table 3). The chromosome had a size of 4,718,403 bp coding for 4,398 predicted proteins and a GC-content of 50.8%. The ST of EC-JS316 was 410, which has been associated with international high-risk clones and acquisition of ESBL as well as different carbapenemases including OXA-181 (Roer et al., 2018; Patiño-Navarrete et al., 2020).

**FIGURE 1:**
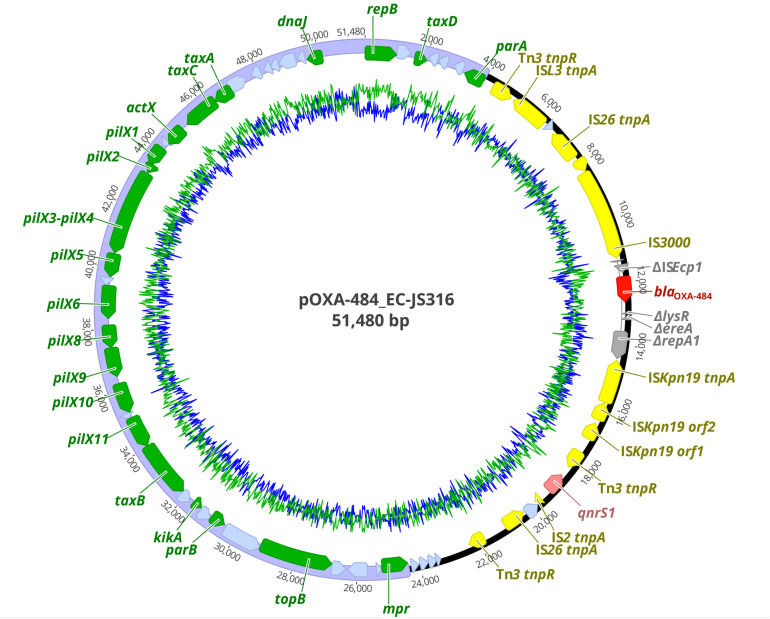
Plasmid map of pOXA-484 harboring *bla*_OXA__–__484_. Colored arrows indicate antibiotic resistance genes *bla*_OXA__–__484_ (dark red) and *qnr*S1 (light red), insertion elements (yellow), disrupted genes (gray), replication, partitioning, plasmid stabilization, and transfer genes of the IncX3 plasmid backbone (green) and hypothetical proteins (light blue). The IncX3 plasmid backbone is marked in purple. GC content is represented by the inner green line and AT content by the inner blue line.

The resistome of EC-JS316 comprised the β-lactamase genes *bla*_OXA__–__484_, *bla*_TEM__–__1_, and *bla*_EC__–__15_. *Bla*_OXA__–__484_ was localized together with the fluoroquinolone resistance gene *qnr*S1 on the IncX3 plasmid pOXA-484 with a size of 51,480 bp (Figure 1). Additional antibiotic resistance genes were localized on the chromosome (*bla*_EC__–__15_), on a 67 kb IncF plasmid and a non-typeable 9 kb plasmid, conferring resistance to trimethoprim-sulfamethoxazole (*dfr*A17, *sul*2), aminoglycosides [*aph*(6)-Id, *aac*(3)-IId, *aad*A5], tetracyclines [*tet*(B)], and macrolides [*mph*(A)] (Supplementary Table 3).

### Sequence Analysis of pOXA-484

The plasmid pOXA-484 consisted of a conserved IncX3 backbone region of ∼31 kb length encoding 21 genes for plasmid replication, mobilization and stabilization as well as 19 hypothetical proteins (Figure 1). The second ∼20 kb region of the plasmid contained the two antibiotic resistance genes *bla*_OXA__–__484_ and *qnr*S1 inside a mosaic region of 12 insertion sequences, 6 genes coding for hypothetical proteins, and 4 disrupted genes (Figure 1). Two IS*26* sequences form a functional composite transposon including the gene *bla*_OXA__–__484_ (Qin et al., 2018). The *bla*_OXA__–__484_ was flanked by a truncated IS*Ecp1* element and two AT-rich direct target repeats (ATCTT), which has been associated with mobilization of *bla*_OXA__–__181_ from ColE2 to IncX3 plasmids (Liu et al., 2015).

For sequence comparison of pOXA-484 to plasmids encoding related OXA-48-like variants, we conducted whole genome sequencing (WGS) using short- and long-read sequencing of the clinical isolates *E. coli* 2800 (EC-2800) and *K. pneumoniae* 4814 (KP-4814). EC-2800 carried *bla*_OXA__–__181_ on an IncX3 plasmid of 51.5 kb and KP-4814 *bla*_OXA__–__232_ on a ColE plasmid of 6.1 kb. Both plasmids share high sequence similarity of >99.9% with previously described plasmids encoding *bla*_OXA__–__181_ and *bla*_OXA__–__232_ (Qin et al., 2018; Li et al., 2019).

**FIGURE 2:**
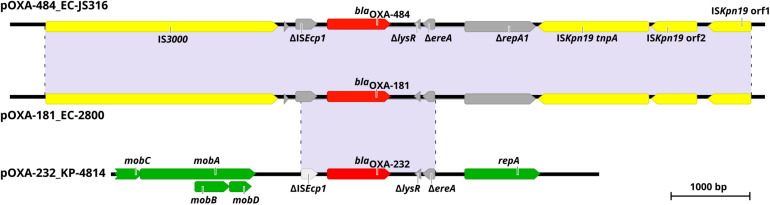
Genetic environment of *bla*_OXA__–__484_, *bla*_OXA__–__181_, and *bla*_OXA__–__232_ within pOXA-484_EC-JS316, pOXA-181_EC-2800, and pOXA-232_KP-4814. Colored arrows indicate insertion elements (yellow), fragmented genes (gray), antibiotic resistance genes (red) and genes of the plasmid backbone (green). Shaded regions between the plasmids share >99% nucleotide sequence identity.

Comparison of pOXA-484 to the nucleotide sequence of pOXA-181 from EC-2800 revealed a sequence identity of >99.9% with only four bases difference (Figure 2). Sequence comparison of pOXA-484 to the NCBI database revealed numerous IncX3 plasmids harboring *bla*_OXA__–__181_ from different *Enterobacterales* with a nucleotide sequence similarity >99.9% (accession no. KX523903.1, MG893567.1, and MK412916.1) indicating a common origin (Skalova et al., 2017; Qin et al., 2018; Mouftah et al., 2019). The sequence comparison of pOXA-484 to pOXA-232 from isolate KP-4814 revealed a fragment of only 2,964 bp shared between the two plasmids with >99.9% sequence identity (Figure 2).

### Horizontal Gene Transfer of pOXA-484

To evaluate the HGT efficiency of pOXA-484, we compared the transconjugation frequencies of plasmid pOXA-484 carrying *bla*_OXA__–__484_ to the frequencies of plasmids encoding *bla*_OXA__–__48_, *bla*_OXA__–__181_, and *bla*_OXA__–__232_, using the recipient strains *E. coli* J53 and *K. quasipneumoniae* subsp. *quasipneumoniae* PRZ. Four representative clinical isolates carrying *bla*_OXA__–__48_ or *bla*_OXA__–__181_ were chosen from a collection of clinical isolates based on species (*E. coli* and *K. pneumoniae*) and different STs. Since OXA-232 is rarely found in *E. coli*, only two clinical *K. pneumoniae* isolates harboring *bla*_OXA__–__232_ were selected (Table 1 and Supplementary Table 4).

**FIGURE 3:**
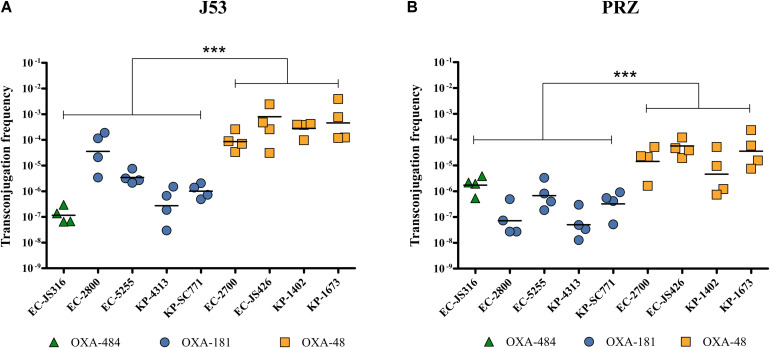
Transconjugation frequencies during horizontal transfer of plasmids harboring *bla*_OXA__–__484_, *bla*_OXA__–__181_, and *bla*_OXA__–__48_ from *E. coli* and *K. pneumoniae* clinical isolates to *E. coli* J53 and *K. quasipneumoniae* subsp. *quasipneumoniae* PRZ. Each dot represents the frequency of a single transconjugation experiment, whereas the bars indicate the mean of all experiments. ***P < 0.0001 (Mann-Whitney *U* test). Transconjugation frequencies to **(A)** *E. coli* J53 and **(B)** *K. quasipneumoniae* subsp. *quasipneumoniae* PRZ.

Transconjugation of plasmids harboring *bla*_OXA__–__48_, *bla*_OXA__–__181_, and *bla*_OXA__–__484_ from all clinical isolates to *E. coli* J53 and *K. quasipneumoniae* subsp. *quasipneumoniae* PRZ was observed, thereby showing intraspecies and intergenus HGT (Figure 3). Transconjugation of plasmid pOXA-484 to J53 and PRZ occurred at mean frequencies of 1.4 × 10^–7^ and 2.1 × 10^–6^, respectively (Figure 3). Similar frequencies were determined for *bla*_OXA__–__181_, ranging from 2.7 × 10^–7^ to 3.6 × 10^–5^ for J53 and 5.2 × 10^–8^ to 6.7 × 10^–7^ for PRZ. In contrast, transconjugation frequencies of *bla*_OXA__–__48_ from *E. coli* and *K. pneumoniae* donors to J53 and PRZ were significantly higher, ranging from 8.6 × 10^–5^ to 8.0 × 10^–4^ for J53 and 1.3 × 10^–5^ to 3.6 × 10^–5^ for PRZ (*P* < 0.001).

*E. coli* J53 Tc were further investigated by long-read genome sequencing and disk diffusion which verified the presence of the respective *bla*_OXA__–__48__–__like_ as well as *qnrS1* in *bla*_OXA__–__181_ and the *bla*_OXA__–__484_ Tc, respectively, as anticipated (Supplementary Tables 5, 6). For *bla*_OXA__–__181_ and *bla*_OXA__–__484_ Tc only the *bla*_OXA__–__48_-like harboring IncX3 plasmids were transferred. Notably, in three out of four *bla*_OXA__–__48_ Tc additional plasmids were identified: a 61.1 kb IncFII plasmid in Tc-EC-2700, a 5.2 kb Col440II plasmid in Tc-EC-1402 and a 3.5 kb Col(pHAD28) as well as a 10.7 kb Col440II plasmid in Tc-EC-1673. However, these plasmids did not harbor any antibiotic resistance genes nor was an impact on HGT frequency or antibiotic resistance phenotype observed when comparing all *bla*_OXA__–__48_ Tc to each other (Figure 3 and Supplementary Table 6).

### Beta-Lactam Resistance Mediated by OXA-484

To analyze the resistance phenotype caused by OXA-484, we evaluated MICs of Tc or Tf harboring the natural plasmids as well as Tf carrying pTOPO expression vectors encoding the genes for OXA-484, OXA-181, OXA-232, OXA-244, and OXA-48, respectively. Since *bla*_OXA__–__244_ is generally localized on the chromosome, only MICs of Tf carrying pTOPO expression vector encoding *bla*_OXA__–__244_ were analyzed.

The presence of pOXA-48-like plasmids resulted in increased MICs for penicillins and carbapenems compared to J53 and PRZ parental strains, whereas only minor differences were observed for cephalosporins and aztreonam (Table 2). Carbapenem MICs of pOXA-484 were either unchanged or slightly lower compared to other OXA-48 variants. In contrast, differences in MICs of at least 2-fold between the OXA-48-like variants were detected for the antibiotics piperacillin ± tazobactam and temocillin. Tc with plasmids encoding *bla*_OXA__–__48_ and *bla*_OXA__–__181_ presented higher MICs for temocillin compared to recipients harboring *bla*_OXA__–__484_ and *bla*_OXA__–__232_ but lower MICs for piperacillin ± tazobactam in case of J53.

In J53 and PRZ Tf carrying the high copy pTOPO expression vector, the lower MICs for temocillin caused by OXA-484 and OXA-232 compared to OXA-181 and OXA-48 were also observed. Transformants carrying TOPO encoding *bla*_OXA__–__244_ showed almost identical MICs for all antibiotics tested compared to Tf pTOPO OXA-484. Furthermore, pTOPO OXA-48 Tf displayed higher carbapenem MICs compared to the other Tf.

Taken together, the β-lactam resistance patterns of Tc and Tf harboring *bla*_OXA__–__484_ revealed similar MICs to OXA-232 and OXA-244. Compared to OXA-181 and OXA-48, piperacillin ± tazobactam MICs were higher for Tc pOXA-484 J53, whereas temocillin MICs were lower for both Tc and Tf harboring either the natural plasmid or pTOPO expression vector.

## Discussion

This study is the first characterization of the carbapenemase OXA-484, a variant of the growing family of OXA-48-like enzymes. The dissemination of OXA-48-like carbapenemases has been attributed to highly efficient HGT to different species, low fitness burden of plasmids encoding *bla*_OXA__–__48__–__like_, association with epidemiological successful lineages and difficult to detect carbapenemase phenotypes (Hamprecht et al., 2019; Pitout et al., 2019). The plasmid pOXA-484 from isolate EC-JS316 was transferable to *E. coli* and *K. quasipneumoniae* subsp. *quasipneumoniae*, revealing efficient intraspecies and intergenus transfer (Figure 3). The transconjugation frequencies of this plasmid are very similar to those of the widely distributed IncX3 plasmid harboring *bla*_OXA__–__181_. Sequence analysis of pOXA-484 revealed the close relationship to IncX3 plasmids containing *bla*_OXA__–__181_ suggesting that pOXA-484 might has evolved from pOXA-181 (Figure 2). Sequence analysis of IncX3 plasmids harboring *bla*_OXA__–__181_ has revealed the potential mobilization of *bla*_OXA__–__181_ by action of an IS*Ecp1* from ColE-plasmids to IncX3 plasmids (Liu et al., 2015). The highly similar sequences between the IncX3 plasmids harboring *bla*_OXA__–__484_ and *bla*_OXA__–__181_ including the IS*Ecp1* elements and two AT-rich direct target repeats bracketing *bla*_OXA__–__484_ suggest the same mechanism of mobilization for *bla*_OXA__–__484_. The spread of OXA-181 and OXA-232 has additionally been associated with specific high-risk clones like ST101, ST307 and ST15 for *K. pneumoniae* and ST38 and ST410 for *E. coli* (Cubero et al., 2015; Li et al., 2019; Pitout et al., 2019; Chudejova et al., 2021). Notably, the *E. coli* isolate JS316 belongs to the high-risk ST410, which has been identified as a worldwide distributed extraintestinal pathogenic *E. coli* lineage causing nosocomial outbreaks (Roer et al., 2018; Patiño-Navarrete et al., 2020).

The β-lactam resistance pattern caused by OXA-484 was comparable to the resistance phenotypes mediated by OXA-232 and OXA-244. However, carbapenem and temocillin MICs in OXA-484 expressing *E. coli* and *K. quasipneumoniae* subsp. *quasipneumoniae* were lower compared to OXA-48 and OXA-181 producers (Tables 2, 3). In a recent study, analyses of kinetic parameters and structural models of OXA-181 variants with mutations at amino acid position 214 in the β5-β6 loop revealed the critical impact of this amino acid on β-lactamase activity (Supplementary Figure 1) (Oueslati et al., 2020). Interestingly, an OXA-181 variant with a R214G mutation resembling the exact amino acid sequence of OXA-484 was included in this study. Enzyme kinetics of OXA-181 214G (or OXA-484) revealed low catalytic efficiencies for temocillin [0.15 ± 0.07 k_cat_/K_*m*_ (mM^–1^/s^–1^)] and imipenem [20 ± 6.9 k_cat_/K_m_ (mM^–1^/s^–1^)], which are similar to OXA-232 and lower than for OXA-181 and OXA-48 (Oueslati et al., 2014, 2020). These results are in line with our antimicrobial susceptibility results (Tables 2, 3).

**TABLE 3:** MICs of *E. coli* J53 and *K. quasipneumoniae* subsp. *quasipneumoniae* PRZ transformants (Tf) harboring pTOPO plasmids encoding *bla*_OXA__–__484_, *bla*_OXA__–__232_, *bla*_OXA__–__244_, *bla*_OXA__–__181_, and *bla*_OXA__–__48_.

	MIC (mg/L)											
β-Lactam^a,b,c^	J53	Tf pTOPO OXA-484 J53	Tf pTOPO OXA-232 J53	Tf pTOPO OXA-244 J53	Tf pTOPO OXA-181 J53	Tf pTOPO OXA-48 J53	PRZ	Tf pTOPO OXA-484 PRZ	Tf pTOPO OXA-232 PRZ	Tf pTOPO OXA-244 PRZ	Tf pTOPO OXA-181 PRZ	Tf pTOPO OXA-48 PRZ
Amoxicillin+CLA	2	>256	>256	>256	>256	>256	2	>256	>256	>256	>256	>256
Ampicillin+SUL	2	>256	>256	>256	>256	>256	8	>256	>256	>256	>256	>256
Piperacillin+TAZ	1	>256	>256	>256	>256	>256	8	>256	>256	>256	>256	>256
Ticarcillin+CLA	4	>256	>256	>256	>256	>256	16	>256	>256	>256	>256	>256
Cefuroxime	4	32	32	16	32	32	4	16	16	8	16	16
Cefotaxime	0.06	1	1	1	1	2	0.06	1	1	0.5	1	1
Ceftazidime	0.06	0.5	0.5	0.5	0.5	0.5	0.03	0.5	0.5	0.5	0.5	0.5
**Temocillin**	4	**128**	**128**	**128**	>**1,024**	>**1,024**	2	**512**	**512**	**512**	>**1,024**	>**1,024**
Ertapenem	0.03	2	2	1	2	4	0.01	2	2	2	2	4
**Imipenem**	0.25	2	2	1	2	4	0.12	**1**	**1**	**1**	**2**	**4**
Meropenem	0.03	1	1	0.5	1	2	0.03	1	1	1	2	2
Aztreonam	0.03	0.5	0.5	0.25	0.25	0.5	0.01	0.25	0.12	0.06	0.12	0.12

**^*a*^CLA, clavulanic acid (2 mg/L). ^*b*^SUL, sulbactam (4 mg/L). ^*c*^TAZ, tazobactam (4 mg/L). MICs of Tf with at least 2-fold differences compared to OXA-484 are highlighted in bold.**

Substitution of arginine at amino acid 214 in OXA-48 to glycine in OXA-244 (R214G), which is also found in OXA-484, led to a reduced hydrolysis activity and MICs for temocillin and carbapenems (Hoyos-Mallecot et al., 2017). Reduced temocillin hydrolysis will most likely affect the sensitivity of temocillin-based screening plates and tests (Hopkins et al., 2019). Recently, increasing numbers of isolates expressing OXA-244 in the European Union have been linked with difficult detection because of only moderately elevated MICs for carbapenems of these isolates (European Centre for Disease Prevention and Control, 2020; Kremer et al., 2020). Likewise, the isolate EC-JS316 harboring *bla*_OXA__–__484_ did not grow on selective agar plates used for detection of CPE, suggesting that OXA-484 might be more easily missed and hence be more prevalent than currently known.

IncX3 plasmids have no significant negative effect on fitness of *Enterobacterales* isolates, which has also been shown for highly successful plasmids of the IncL group harboring *bla*_OXA__–__48_ and which is a predictor for efficient dissemination (Kassis-Chikhani et al., 2013; Hamprecht et al., 2019). These properties of the IncX3 plasmids might promote the further distribution of OXA-484, as already shown for other carbapenemases like NDM or KPC as well as CTX-M type ESBLs (Kassis-Chikhani et al., 2013; Wu et al., 2019).

## Data Availability Statement

The datasets presented in this study can be found in online repositories. The names of the repository/repositories and accession number(s) can be found below: https://www.ncbi.nlm.nih.gov/bioproject/PRJNA644256 and https://www.ncbi.nlm.nih.gov/bioproject/PRJNA644257.

## Ethics Statement

All bacterial strains were isolated as part of routine microbiological diagnostics and stored in an anonymized database. According to the Ethics Committee of the Hospital of Johann Wolfgang Goethe-University, Frankfurt am Main, no informed consent or ethical approval of the study is necessary.

## Author Contributions

JSo and SG designed the study, analyzed the data, and drafted the manuscript. JSo, FK, KG, FW, MT, and SR-C performed the experiments. JSo carried out the sequencing and bioinformatic analyses. JSa, AH, and VK edited the manuscript. All authors reviewed the manuscript.

## Conflict of Interest

The authors declare that the research was conducted in the absence of any commercial or financial relationships that could be construed as a potential conflict of interest.

## Footnotes

1 https://github.com/tseemann/mlst

2 https://github.com/tseemann/abricate

3 https://geneious.com
