# Supplementary material for: OXA-484, an OXA-48-Type Carbapenem-Hydrolyzing Class D β-Lactamase From Escherichia coli
Source: Front Microbiol. 2021 May 12;12:660094. doi: 10.3389/fmicb.2021.660094 (PMC8153228; doi:10.3389/fmicb.2021.660094)
Supplement: Supplementary file 1 [file Table_1.docx]

OXA-484, an OXA-48-type carbapenem-hydrolyzing class D β‑lactamase from *Escherichia coli*

Julian Sommer, Kristina M. Gerbracht, Felix F. Krause, Florian Wild, Manuela Tietgen, Sara Riedel-Christ, Janko Sattler, Axel Hamprecht, Volkhard A. J. Kempf, Stephan Göttig

Supplementary Material

**Supplementary Table 1** Primers used for PCR amplification of open reading frames and promoters of *bla*_OXA-484_, *bla*_OXA-244_, *bla*_OXA-232_, *bla*_OXA-181_ and *bla*_OXA-48_. Each reaction was performed using the corresponding TOPO-F1 forward primer for the respective *bla*_OXA-48_ variant and preOXA-48B as reverse primer. The annealing temperature for all reactions was 60°C.

| **Primer** | **Target** | **Direction** | **Sequence 5´🡪 3´** | **Reference** |
| --- | --- | --- | --- | --- |
| OXA-TOPO-F1 | *bla*_OXA-484_, *bla*_OXA-232_, *bla*_OXA-181_ | forward | AGTGTTGCTCTGTGGATAAC | This study |
| OXA-244-TOPO-F1 | *bla*_OXA-244_ | forward | TGTTTTACGGCAGTGAGAGC | This study |
| OXA-48-TOPO-F1 | *bla*_OXA-48_ | forward | ATTGGTAGACAGCTAACCGTTCTTG | This study |
| preOXA-48B | *bla*_OXA-484_, *bla*_OXA-232_, *bla*_OXA-181_*,* *bla*_OXA-244_*, bla*_OXA-48_ | reverse | CACACAAATACGCGCTAACC | (Potron et al., 2011) |

**Supplementary Table 2** MICs were determined by antibiotic gradient strips, microdilution in case of colistin and agar dilution for fosfomycin as recommended by EUCAST. Beta-lactamase inhibitors were used with fixed concentrations as recommended by EUCAST: clavulanic acid (CLA; 2 mg/L), sulbactam (SUL; 4 mg/L), tazobactam (TAZ; 4 mg/L), avibactam (AVI; 4 mg/L).

| **Antibiotic** | **EC-JS316 MIC (mg/L)** |
| --- | --- |
| Ampicillin + SUL | >256 |
| Piperacillin + TAZ | >256 |
| Ticarcillin + CLA | >256 |
| Cefuroxime | >256 |
| Cefoxitin | 64 |
| Cefotaxime | 4 |
| Cefepime | 1 |
| Ceftazidime | 2 |
| Ceftazidime + AVI | 0.5 |
| Temocillin | 128 |
| Ertapenem | 1 |
| Imipenem | 0.25 |
| Meropenem | 0.12 |
| Doripenem | 0.06 |
| Aztreonam | 1 |
| Ciprofloxacin | >32 |
| Levofloxacin | >32 |
| Gentamicin | 64 |
| Amikacin | 2 |
| Tobramycin | 8 |
| Tigecycline | 0.5 |
| Fosfomycin | 1 |
| Trimethoprim-sulfamethoxazole | >32 |
| Colistin | 0.5 |

**Supplementary Table 3** Characteristics of the circular chromosome and circular plasmids of the clinical isolate EC-JS316 harboring *bla*_OXA-484_.

| **Chromosome/Plasmid** | **Size (bp)** | **Inc type** | **Resistance genes** | **Accession no.** |
| --- | --- | --- | --- | --- |
| Chromosome | 4,718,403 | Not applicable | *bla*_EC-15_ | CP058618.1 |
| Plasmid 1 | 91,318 | Non-typeable | None | CP058619.1 |
| Plasmid 2 | 67,321 | IncF [F48:A1:B49] | *bla*_TEM-1_, *tet*(B)*, aph*(6)-Id*, aph*(3'')-IId*, sul2, aac(3)* | CP058620.1 |
| Plasmid 3 | 51,480 | IncX3 | *bla*_OXA-484_*, qnr*S1 | CP058621.1 |
| Plasmid 4 | 9,135 | Non-typeable | *aad*A5*, dfr*A17*, mph*(A) | CP058622.1 |
| Plasmid 5 | 1,551 | Col(MG828) | None | CP058623.1 |

| **Isolate** | **Species** | **Size/bp** | **N50** | **GC %** | **Contigs** | **Beta-lactamase (*bla*) genes encoding** | **Additional antibiotic resistance genes** | **Accession no.** |
| --- | --- | --- | --- | --- | --- | --- | --- | --- |
| EC-JS316 | *E. coli* | 4,939,208 | 4,718,403 | 50.65 | 6 | OXA-484, EC-15, TEM-1 | *aac(3), aadA5, aph(3'')-IId, aph(6)-Id, dfrA17, mph(A), sul2, tet(B), qnrS1* | CP058618:CP058623 |
| EC-2700 | *E. coli* | 5,389,695 | 5,146,710 | 50.47 | 7 | OXA-48, CMY-42 | *aph(3'')-Ib, aph(6)-Id, catA2, mdf(A), mph(A), tet(A)* | JAGFZN000000000 |
| EC-JS426 | *E. coli* | 5,036,724 | 4,830,935 | 50.86 | 3 | OXA-48, TEM-1 | *aadA2, ant(3'')-Ia, aph(3'')-Ia, aph(3'')-Ib, aph(6)-Id, dfrA14, floR, mdf(A), mph(A), sul3, tet(A)* | CP072322:CP072324 |
| KP-1402 | *K. pneumoniae* | 5,563,514 | 5,217,503 | 57.30 | 5 | OXA-48, CTX-M-15, TEM-1B, SHV-145, OXA-1 | *aac(6')-Ib-cr, aadA2, dfrA12, fosA5, mph(A), oqxA, oqxB, sul1, tet(A)* | JAGFZO000000000 |
| KP-1673 | *K. pneumoniae* | 5,535,002 | 5,394,767 | 57.01 | 4 | OXA-48, CTX-M-15, TEM-1A, SHV-106, OXA-1, OXA-9, | *aac(3)-Iia, aac(6')-Ib-cr, ant(3'')-Ia, dfrA14, fosA, tet(D)* | JAGFZP000000000 |
| KP-4113 | *K. pneumoniae* | 5,602,773 | 1,932,407 | 57.11 | 22 | OXA-232, CTX-M-15, TEM-1B, SHV-106, SHV-12 | *aadA2, arr-3, catA1, dfrA12, erm(B), fosA, mph(A), oqxA, rmtF, sul1, tet(B), qepA4* | JAGFZQ000000000 |
| KP-4814 | *K. pneumoniae* | 5,810,700 | 3,919,546 | 56.74 | 6 | OXA-232, CTX-M-15, TEM-1A, SHV-106, OXA-1 | *aadA2, aac(6')-Ib-cr, armA, dfrA1, dfrA12, dfrA14, fosA6, mph(E), msr(E), sul1, tet(D), oqxA, oqxB* | JAGGIL000000000 |
| EC-5255 | *E. coli* | 5,386,534 | 4,966,358 | 50.63 | 9 | OXA-181, CTX-M-15, TEM-1B | *aph(3'')-Ib, aph(6)-Id, dfrA14, mdf(A), sul2, tet(A), qnrS1* | JAGFZR000000000 |
| EC-2800 | *E. coli* | 4,931,200 | 3,156,159 | 50.72 | 8 | OXA-181, TEM-35, OXA-1 | *ant(3'')-Ia, aph(3'')-Ib, aph(6)-Id, catA1, dfrA1, mdf(A), sul2, tet(B), qnrS1* | JAGDMO010000000 |
| KP-4313 | *K. pneumoniae* | 5,482,385 | 5,209,357 | 57.72 | 8 | OXA-181, CTX-M-3, OKP-B-1 | *fosA, oqxA, oqxB, qnrS1* | JAGFZS000000000 |
| KP-SC771 | *K. pneumoniae* | 5,601,047 | 5,292,628 | 57.18 | 6 | OXA-181, CTX-M-15, TEM-206, SHV-106, OXA-1 | *aac(3)-Iia, aac(6')-Ib-cr, aph(3'')-Ib, aph(6)-Id, dfrA14, fosA6, oqxA, oqxB, sul2, tet(A), qnrB1, qnrS1* | JAGFZT000000000 |
| EC-SC1140 | *E. coli* | 5,458,071 | 5,289,836 | 50.47 | 2 | OXA-244, CTX-M-14b | *ant(3'')-Ia, dfrA1, mdf(A)* | JAGFZU000000000 |

**SUPPLEMENTARY TABLE 4** Genetic characteristics of OXA-48-like producing *E. coli* and *K. pneumoniae* isolates. Assembly statistics were calculated using the QUAST v5.0.2 (Gurevich et al., 2013).

**SUPPLEMENTARY TABLE 5** Genetic characteristics of transconjugants harboring plasmids with *bla*_OXA-48-like_ resistance genes and the recipient *E. coli* J53 without plasmids. The strains were sequenced using a MinION MK1B sequencer (Oxford Nanopore Technologies, Oxford, UK) to a minimal mean coverage of 25x and reads were filtered by length (>1000 bp) and phred quality score (>7) using NanoFilt (version 2.8.0) (De Coster et al., 2018). Assemblies were conducted utilizing Flye (version 2.8.3), and contigs were polished using rebaler^1^ (version 0.2.0) and Medaka^2^ (version 0.10.0) (Kolmogorov et al., 2019). ABRicate^3^ (version 1.0.1) was applied using the databases CGE Plasmid Finder and NCBI AMRFinderPlus for identification of plasmid incompatibility groups and antibiotic resistance genes respectively, using thresholds of 98% gene coverage and ≥ 98 % nucleotide sequence identity. Assembly statistics were calculated using QUAST (version 5.0.2) (Gurevich et al., 2013).

^1^https://github.com/rrwick/Rebaler ^2^https://github.com/nanoporetech/medaka ^3^https://github.com/tseemann/abricate

| **Strain** | **Donor** | **Size/bp** | **N50** | **GC %** | **Contigs** | **ST** | **Antibiotic resistance genes** | **Plasmid types** |
| --- | --- | --- | --- | --- | --- | --- | --- | --- |
| Tc-EC-JS316 | EC-JS316 | 4,684,364 | 310,496 | 50.89 | 30 | 10 | *bla*_OXA-484_*, qnrS1, mdf(A)* | IncX3 |
| Tc-EC-2700 | EC-2700 | 4,739,131 | 394,629 | 50.91 | 25 | 10 | *bla*_OXA-48_*, mdf(A)* | IncL, IncFII |
| Tc-EC-JS426 | EC-JS426 | 4,744,014 | 3848515 | 50.78 | 5 | 10 | *bla*_OXA-48_*, mdf(A)* | IncL |
| Tc-KP-1402 | KP-1402 | 4,567,039 | 195,902 | 51.09 | 42 | 10 | *bla*_OXA-48_*, mdf(A)* | IncL. Col440II |
| Tc-KP-1673 | KP-1673 | 4,766,648 | 2,862,871 | 50.79 | 12 | 10 | *bla*_OXA-48_*, mdf(A)* | IncL, Col(pHAD28), Col440II |
| Tc-EC-5255 | EC-5255 | 4,784,966 | 885,466 | 50.82 | 9 | 10 | *bla*_OXA-181_*, qnrS1, mdf(A)* | IncX3 |
| Tc-EC-2800 | EC-2800 | 4,702,790 | 829,684 | 50.73 | 9 | 10 | *bla*_OXA-181_*, qnrS1, mdf(A)* | IncX3 |
| Tc-KP-4313 | KP-4313 | 4,738,327 | 4,688,081 | 50.71 | 2 | 10 | *bla*_OXA-181_*, qnrS1, mdf(A)* | IncX3 |
| Tc-KP-SC771 | KP-SC771 | 4,733,264 | 4,684,076 | 50.72 | 2 | 10 | *bla*_OXA-181_*, qnrS1, mdf(A)* | IncX3 |
| J53 | - | 4,684,071 | 4,684,071 | 50.77 | 1 | 10 | *mdf(A)* | none |

**SUPPLEMENTARY TABLE 6** Disk diffusion zone diameters in mm of transconjugants of recipients J53 and PRZ harboring pOXA-484, pOXA‑181 or pOXA-48 and the recipients without resistance plasmids. Disk diffusion was conducted as recommended by EUCAST. CLA, clavulanic acid; SUL, sulbactam; TAZ, tazobactam.

|  |  | **J53 transconjugants** | | | | | | | | |  | **PRZ transconjugants** | | | | | | | | |
| --- | --- | --- | --- | --- | --- | --- | --- | --- | --- | --- | --- | --- | --- | --- | --- | --- | --- | --- | --- | --- |
| **Antibiotic** | J53 | EC-JS316 | EC-2700 | EC-JS426 | KP-1402 | KP-1673 | EC-5255 | EC-2800 | KP-4313 | KP-SC771 | PRZ | EC-JS316 | EC-2700 | EC-JS426 | KP-1402 | KP-1673 | EC-5255 | EC-2800 | KP-4313 | KP-SC771 |
| Ampicillin | 20 | 6 | 6 | 6 | 6 | 6 | 6 | 6 | 6 | 6 | 6 | 6 | 6 | 6 | 6 | 6 | 6 | 6 | 6 | 6 |
| Amoxicillin+CLA | 24 | 6 | 6 | 6 | 6 | 6 | 6 | 6 | 6 | 6 | 20 | 6 | 6 | 6 | 6 | 6 | 6 | 6 | 6 | 6 |
| Ampicillin+SUL | 22 | 6 | 6 | 6 | 6 | 6 | 6 | 6 | 6 | 6 | 20 | 6 | 6 | 6 | 6 | 6 | 6 | 6 | 6 | 6 |
| Piperacillin | 30 | 14 | 10 | 10 | 10 | 10 | 12 | 10 | 10 | 10 | 20 | 6 | 6 | 6 | 6 | 6 | 6 | 6 | 6 | 6 |
| Piperacillin+TAZ | 28 | 14 | 10 | 10 | 10 | 10 | 10 | 10 | 10 | 10 | 24 | 6 | 6 | 6 | 6 | 6 | 6 | 6 | 6 | 6 |
| Cefuroxime | 24 | 24 | 24 | 22 | 22 | 22 | 24 | 22 | 24 | 22 | 22 | 20 | 20 | 20 | 20 | 20 | 20 | 20 | 20 | 22 |
| Cefoxitin | 28 | 26 | 26 | 24 | 24 | 24 | 24 | 24 | 24 | 24 | 22 | 20 | 22 | 22 | 20 | 20 | 20 | 20 | 20 | 22 |
| Cefotaxime | 28 | 26 | 26 | 24 | 24 | 24 | 24 | 24 | 26 | 24 | 24 | 22 | 23 | 20 | 20 | 20 | 20 | 20 | 20 | 20 |
| Ceftazidime | 30 | 28 | 30 | 28 | 30 | 28 | 28 | 28 | 28 | 28 | 28 | 24 | 26 | 24 | 26 | 26 | 24 | 24 | 24 | 24 |
| Temocillin | 24 | 12 | 6 | 6 | 6 | 6 | 6 | 6 | 6 | 6 | 26 | 12 | 6 | 6 | 6 | 6 | 6 | 6 | 6 | 6 |
| Ertapenem | 40 | 26 | 24 | 22 | 24 | 24 | 22 | 22 | 24 | 24 | 34 | 18 | 18 | 18 | 18 | 18 | 16 | 16 | 16 | 18 |
| Imipenem | 38 | 26 | 24 | 22 | 24 | 24 | 22 | 22 | 24 | 22 | 30 | 22 | 22 | 20 | 22 | 20 | 22 | 22 | 20 | 20 |
| Meropenem | 38 | 28 | 26 | 26 | 28 | 28 | 26 | 28 | 26 | 26 | 30 | 22 | 24 | 20 | 22 | 22 | 20 | 20 | 20 | 22 |
| Doripenem | 32 | 24 | 24 | 26 | 26 | 26 | 28 | 26 | 24 | 24 | 30 | 18 | 20 | 20 | 22 | 22 | 20 | 20 | 20 | 20 |
| Aztreonam | 38 | 34 | 34 | 36 | 36 | 36 | 36 | 36 | 34 | 34 | 30 | 30 | 30 | 30 | 30 | 30 | 30 | 30 | 30 | 28 |
| Gentamicin | 22 | 24 | 20 | 22 | 20 | 22 | 22 | 22 | 22 | 22 | 20 | 19 | 18 | 18 | 20 | 18 | 20 | 18 | 18 | 18 |
| Amikacin | 22 | 22 | 20 | 20 | 22 | 22 | 22 | 20 | 20 | 20 | 20 | 20 | 18 | 20 | 20 | 22 | 21 | 18 | 20 | 20 |
| Tobramycin | 20 | 20 | 20 | 22 | 22 | 20 | 22 | 20 | 22 | 20 | 20 | 18 | 20 | 18 | 20 | 18 | 18 | 18 | 18 | 18 |
| Trimethoprim/  Sulfamethoxazol | 32 | 32 | 32 | 32 | 32 | 30 | 32 | 30 | 32 | 30 | 6 | 6 | 6 | 6 | 6 | 6 | 6 | 6 | 6 | 6 |
| Ciprofloxacin | 40 | 32 | 40 | 38 | 40 | 40 | 32 | 30 | 32 | 32 | 28 | 20 | 28 | 28 | 26 | 28 | 20 | 18 | 20 | 18 |
| Levofloxacin | 40 | 30 | 40 | 38 | 40 | 38 | 28 | 28 | 28 | 30 | 30 | 20 | 28 | 28 | 28 | 26 | 20 | 20 | 18 | 18 |

**Supplementary Figure 1** Amino acid sequence alignment of the OXA-48-like variants OXA-48, OXA-181, OXA‑232, OXA-244 and OXA-484 using ClustalW and ESPript 3.0. Sequence similarities are shaded in black, differences are highlighted in white. The amino acid residue 214 is marked in red. Secondary structure elements of OXA-181 were received from PDB entry 5OE0 and are illustrated above the sequences (Lund et al., 2017). Phylogeny of proteins is illustrated by the dendrogram based on an alignment of the amino acid sequences. The alignment was generated using MAFFT v7.450 (Katoh and Standley, 2013) and phylogeny was constructed using FastTree 2.1.11 (Price et al., 2010).


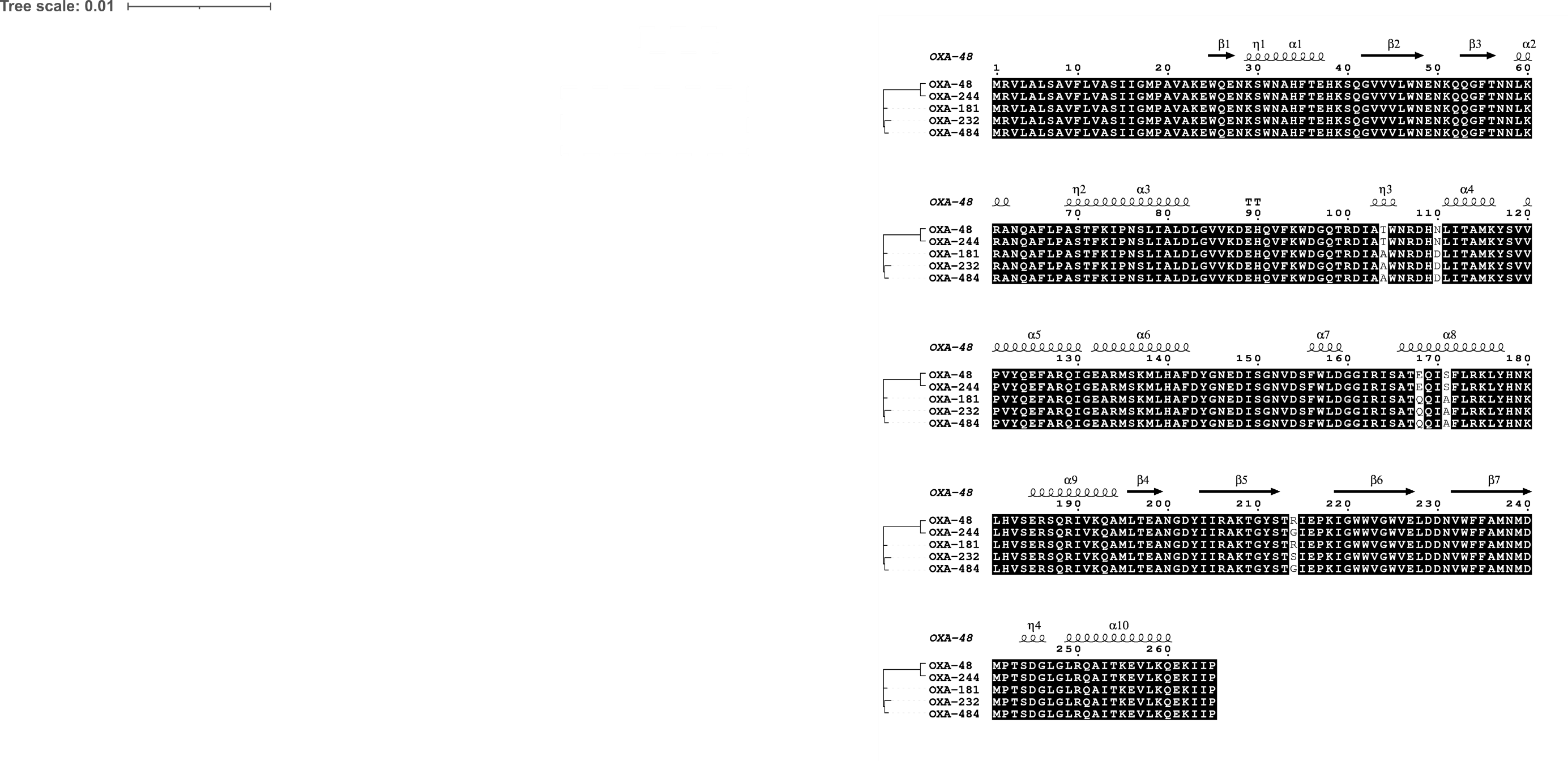


References

De Coster, W., D’Hert, S., Schultz, D. T., Cruts, M., and Van Broeckhoven, C. (2018). NanoPack: Visualizing and processing long-read sequencing data. *Bioinformatics* 34, 2666–2669. doi:10.1093/bioinformatics/bty149.

Gurevich, A., Saveliev, V., Vyahhi, N., and Tesler, G. (2013). QUAST: quality assessment tool for genome assemblies. *Bioinformatics* 29, 1072–1075. doi:10.1093/bioinformatics/btt086.

Katoh, K., and Standley, D. M. (2013). MAFFT multiple sequence alignment software version 7: Improvements in performance and usability. *Mol. Biol. Evol.* 30, 772–780. doi:10.1093/molbev/mst010.

Kolmogorov, M., Yuan, J., Lin, Y., and Pevzner, P. A. (2019). Assembly of long, error-prone reads using repeat graphs. *Nat. Biotechnol.* 37, 540–546. doi:10.1038/s41587-019-0072-8.

Lund, B. A., Thomassen, A. M., Carlsen, T. J. O., and Leiros, H. K. S. (2017). Structure, activity and thermostability investigations of OXA-163, OXA-181 and OXA-245 using biochemical analysis, crystal structures and differential scanning calorimetry analysis. *Acta Crystallogr. Sect. FStructural Biol. Commun.* 73, 579–587. doi:10.1107/S2053230X17013838.

Potron, A., Nordmann, P., Lafeuille, E., Al Maskari, Z., Al Rashdi, F., and Poirel, L. (2011). Characterization of OXA-181, a carbapenem-hydrolyzing class D beta-lactamase from *Klebsiella pneumoniae*. *Antimicrob. Agents Chemother.* 55, 4896–9. doi:10.1128/AAC.00481-11.

Price, M. N., Dehal, P. S., and Arkin, A. P. (2010). FastTree 2 - Approximately maximum-likelihood trees for large alignments. *PLoS One* 5. doi:10.1371/journal.pone.0009490.
